# Resting State Default Mode Network Connectivity, Dual Task Performance, Gait Speed, and Postural Sway in Older Adults with Mild Cognitive Impairment

**DOI:** 10.3389/fnagi.2017.00423

**Published:** 2017-12-21

**Authors:** Rachel A. Crockett, Chun Liang Hsu, John R. Best, Teresa Liu-Ambrose

**Affiliations:** Aging, Mobility, and Cognitive Neuroscience Laboratory, Department of Physical Therapy, Djavad Mowafaghian Centre for Brain Health, University of British Columbia, Vancouver, BC, Canada

**Keywords:** functional connectivity, default mode network, dual task, gait speed, postural sway, mild cognitive impairment

## Abstract

Aging is associated with an increased risk of falling. In particular, older adults with mild cognitive impairment (MCI) are more vulnerable to falling compared with their healthy counterparts. Major contributors to this increased falls risk include a decline in dual task performance, gait speed, and postural sway. Recent evidence highlights the potential influence of the default mode network (DMN), the frontoparietal network (FPN), and the supplementary motor area (SMA) on dual task performance, gait speed, and postural sway. The DMN is active during rest and deactivates during task-oriented processes, to maintain attention and stay on task. The FPN and SMA are involved in top-down attentional control, motor planning, and motor execution. The DMN shows less deactivation during task in older adults with MCI. This lack of deactivation is theorized to increase competition for resources between the DMN and task-related brain regions (e.g., the FPN and SMA), increasing distraction from the task and reducing task performance. However, no study has yet investigated the relationship between the between-network connectivity of the DMN with these regions and dual task walking, gait speed or postural sway. We hypothesized that greater functional connectivity both within the DMN and between DMN–FPN and DMN–SMA, will be associated with poorer performance during dual task walking, slower gait speed, and greater postural sway in older adults with MCI. Forty older adults with MCI were measured on a dual task-walking paradigm, gait speed over a 4-m walk, and postural sway using a sway-meter. Greater within-DMN connectivity was significantly correlated with poorer dual task performance. Furthermore, greater inter-network connectivity between the DMN and SMA was significantly correlated with slower gait speed and greater postural sway on the eyes open floor sway task. Thus, greater resting state DMN functional connectivity may be an underlying neural mechanism for reduced dual task ability, slower gait speed, and greater postural sway, resulting in the increased risk of mobility disability and falling in older adults with MCI.

## Introduction

Walking has long been considered an automated skill required for daily functioning and independent living. In reality, walking requires the complex neural coordination of visual, proprioceptive, and vestibular sensory incoming information (Beurskens and Bock, 2012). Furthermore, walking in everyday life is not performed as a single task since environmental demands require the ability to perform additional cognitive tasks at the same time (i.e., dual task), such as walking while talking (Yuan et al., 2015). Thus, walking depends on higher-order cognitive processes, known as executive functions (Yogev et al., 2008). Research consistently demonstrates that executive functions are important for successful dual task performance (Coppin et al., 2006).

The risk for developing impaired mobility (e.g., slow walking) increases with age (Verghese et al., 2009; Seidler et al., 2010). Falls are a significant consequence of impaired mobility (Fuller, 2000) with the majority of falls occurring during dual task conditions, such as walking while performing a secondary task (Tideiksaar, 1996). Thus, it is hypothesized that falls may not be a result of balance deficits in isolation, but the inability to effectively allocate attention to postural stability in dual task situations (Lajoie et al., 1996). This hypothesis is supported by observations such as older adults who stop walking while engaged in conversation (i.e., less able to dual task) are more likely to fall than those who continue walking (Lundin-Olsson et al., 1997). Recent evidence also highlights the importance of maintaining dual task ability in older adults beyond falls risk; Montero-Odasso et al. (2017) demonstrated that reduced dual task gait performance among older adults with mild cognitive impairment (MCI) was associated with progression to dementia.

MCI is considered the prodromal stage for dementia and is characterized by global brain atrophy and cognitive decline beyond normal aging but that does not impact daily living (Feldman and Jacova, 2005). Older adults with MCI are also found to have a greater decline in physical functioning (Aggarwal et al., 2006) and poorer performance under dual task conditions (Nascimbeni et al., 2015). Consequently, those with MCI are five times as likely to fall compared to cognitively intact older adults (Tinetti et al., 1988). Despite this, knowledge regarding the underlying neural correlates associated with dual task and gait performance in MCI is lacking. A better understanding of the neural basis for impaired dual task and mobility in this population can inform future strategies to prevent the progression of MCI and subsequently reduce the risk of mobility disability and falling.

Current evidence suggests that both the cognitive and motor impairments associated with MCI have a common neurobiological basis (Silbert et al., 2008; Callisaya et al., 2013). Of relevance, aging is characterized by disruptions in the functional connectivity of neural networks that support both cognitive and motor functions. Recent evidence highlights the potential involvement of the default mode network (DMN) and the frontoparietal network (FPN). The DMN is involved in autobiographical memory, memory consolidation, and self-referential thought (Andrews-Hanna et al., 2007; Buckner et al., 2008). It is active during rest and deactivates during task-oriented processes, to maintain attention and stay on task (Raichle et al., 2001). However, the DMN shows less deactivation on task for older adults and those with MCI (Lustig et al., 2003; Mevel et al., 2011). Although findings are inconsistent, it is thought this lack of deactivation may be due to greater resting state DMN functional connectivity (Mevel et al., 2011). It is suggested that as the posterior components of the network are among the first brain regions to be affected by both age and MCI-related atrophy (Buckner et al., 2005; Choo et al., 2010), compensation occurs as connections between the DMN and frontal regions increase (Davis et al., 2008), leading to a net increase in resting state functional connectivity and a lack of deactivation on task. This lack of deactivation while on task is theorized to increase distraction and reduce cognitive performance (Gardini et al., 2014). With age, walking becomes more dependent on executive functioning (Coppin et al., 2006). Therefore a decline in cognitive performance is subsequently hypothesized to also have a detrimental impact on walking in older adults and those with MCI.

The FPN is involved in top-down attentional control and allocation of available neural resources to important cognitive processes (Corbetta, 1998; Scolari et al., 2015), as well as in motor planning and motor execution (Ptak et al., 2017). It has also been associated with dual task walking performance. Compared to normal walking, performance on a walking while talking task was associated with greater functional connectivity between the prefrontal and supplementary motor areas (SMA) of the FPN and sensorimotor network, respectively (Yuan et al., 2015).

Much like the FPN, the SMA is a key structure for the execution and control of voluntary movement, motor planning (Roland et al., 1980; Eccles, 1982), and for maintaining attention on a motor task (Johansen-Berg and Matthews, 2000). Unlike some of the deeper brain structures, functional activation of the SMA can be assessed while walking using imaging techniques such as near infrared spectroscopy. Research using this technique, has found that an increase in activation of the SMA was associated with declines in gait performance under dual task conditions (Lu et al., 2015). Thus, the SMA may play an important role in the maintenance of gait performance during dual task walking.

Research focusing on the relationship between the DMN and the task-related networks responsible for motor functioning is lacking. However, one study did find that greater functional connectivity between the DMN and FPN was associated with reduced performance on a finger-tapping task in older fallers, indicative of an inability to focus attention on the task (Hsu et al., 2014). It is theorized that in older adults, this greater connectivity between the DMN and task-related networks at rest may suggest the DMN is remaining active on task, competing for resources with the task-related networks, and subsequently causing a decline in performance. This competition for resources is likely exacerbated under dual task conditions (Tombu and Jolicœur, 2003) and is hypothesized to underlie the relationship between cognitive and motor functioning decline, leading to an increased risk of falls and mobility disability in older adults and those with MCI. However, to our knowledge, no research has yet investigated the effect of the functional between-network connectivity of the DMN in relation to dual task walking paradigms.

In addition to reduced dual task performance, slower gait speed and increased postural sway are considered major factors contributing to an increased risk of falls. Older adults with MCI were found to have significantly slower gait speed (Eggermont et al., 2010) and greater postural sway (Liu-Ambrose et al., 2008) compared to healthy older adults. Both the SMA and components of the FPN have been associated with having a functional role in controlling gait speed (Harada et al., 2009; Yuan et al., 2015) and maintaining postural stability (Mihara et al., 2008). Therefore, maladaptive connectivity between these regions and the DMN may also negatively impact gait speed and postural stability, further contributing to an increased risk of falling. No research has yet investigated this potential relationship.

Consequently, the primary objective of this study was to investigate the relationship between the functional connectivity within the DMN and the between-network connectivity of the DMN with both the FPN and SMA (i.e., DMN–FPN and DMN–SMA) during a dual task walking paradigm in older adults with MCI. In addition, the secondary objective was to explore the association of within-network and between-network connectivity with gait speed and postural sway.

**FIGURE 1:**
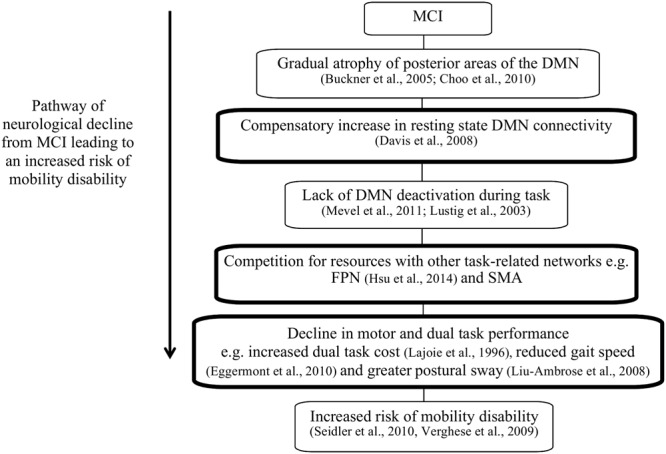
The pathway of neurological decline leading to increased risk of mobility disability in patients with mild cognitive impairment (MCI). The boxes highlighted in black indicate the hypothesized components of this theory that this study aims to investigate.

We hypothesized that greater functional connectivity within the DMN, between DMN–FPN, as well as between the DMN–SMA will be associated with poorer dual task walking performance, slower gait speed and greater postural sway. The rationale and hypotheses for this study are highlighted in **Figure 1**.

## Materials and Methods

### Participants

Forty community dwelling older adults with MCI are included in this cross-sectional study. MCI was defined as: (1) a Montreal Cognitive Assessment (MoCA) score <26/30; (2) have subjective memory complaints (SMC); (3) no significant functional impairment, as determined by a score ≥6/8 on the Lawton and Brody Instrumental Activities of Daily Living Scale; and (4) no dementia.

Participants were recruited from metropolitan Vancouver and interested individuals were telephone screened to confirm general eligibility according to the inclusion and exclusion criteria. We included those who: (1) were aged ≥60 years; (2) scored ≤26/30 on the MoCA (Nasreddine et al., 2005); (3) had SMC, defined as the self-reported feeling of memory worsening with an onset within the last 5 years, as determined by interview (Gifford et al., 2014); (4) preserved general cognition as indicated by a Mini-Mental State Examination (Folstein et al., 1975) score ≥24/30; (5) score ≥6/8 on the Lawton and Brody (Lawton and Brody, 1969) Instrumental Activities of Daily Living Scale; (6) were right hand dominant as measured by the Edinburgh Handedness Inventory (Oldfield, 1971); (7) were living independently in their own homes; (8) had visual acuity of at least 20/40, with or without corrective lenses; and (9) provided informed consent. We excluded those who: (1) had a formal diagnosis of neurodegenerative disease, stroke, dementia (of any type), or psychiatric condition; (2) had clinically significant peripheral neuropathy or severe musculoskeletal or joint disease; (3) were taking psychotropic medication; (4) had a history indicative of carotid sinus sensitivity; (5) were living in a nursing home, extended care facility, or assisted-care facility; or (6) were ineligible for magnetic resonance imaging (MRI) scanning. All participants provided written consent and ethics approval was acquired from the Vancouver Coastal Research Health Institute and University of British Columbia’s Clinical Research Ethics Board.

### Descriptive Variables

Age was quantified in years and education level was assessed by self-report. Standing height was measured as stretch stature to the 0.1 cm per standard protocol. Weight was measured twice to the 0.1 kg on a calibrated digital scale. As previously stated, the MoCA was used as a classification tool for MCI. The MoCA is a 30-point test that covers multiple cognitive domains (Nasreddine et al., 2005). The MoCA has been found to have good internal consistency and test–retest reliability and was able to correctly identify 90% of a large sample of MCI individuals from two different clinics (Nasreddine et al., 2005).

### Dual Task

Participants were also asked to perform a dual task involving a cognitive task while walking. Better performance on dual task measures is associated with better task switching, working memory, and divided attention (Schaefer and Schumacher, 2010). The cognitive task was the serial subtracting sevens task whereby the participant is required to start subtracting sevens aloud from a randomly given number. The walking task was performed on a GAITRite mat (McDonough et al., 2001), which was used to record the time between the first step onto the mat and the last step off of the mat for each task. Participants were instructed to begin walking 1 m before they reached the mat and finish once they made it to 1 m past the end of the mat to control for acceleration and deceleration effects. The participants were first asked to walk three times at a self-selected pace across the mat to give a mean walking time. They then performed the subtracting sevens task standing still (off of the mat) for 30 s. For the dual task component they were asked to walk at a self-selected pace and begin the serial subtraction once on the mat. This was repeated three times to get a mean dual task time. Dual task cost was then calculated by subtracting the mean walking only time from the dual task time, divided by the walking only time [(dual task - walking time)/walking time]. A lower dual task cost score indicated better dual task performance.

### Usual Gait Speed

Participants walked at their usual pace along a 4-m path. To avoid acceleration and deceleration effects, participants started walking 1 m before reaching the 4-m path and completed their walk 1 m beyond it.

Usual gait speed (m/s) was calculated from the mean of two trials. The test–retest reliability of usual gait speed in our laboratory is 0.95 (ICC; Liu-Ambrose et al., 2006).

### Postural Sway

Balance was assessed using the eyes open floor and foam sway component of the Physiological Profile Assessment© 18 (Prince of Wales Medical Research Institute, Randwick, Sydney, NSW, Australia; Lord et al., 2003). Participants were asked to stand with their feet hip width apart and look straight ahead for 30 s, first on the hard floor and secondly on a 3-inch high-density foam cushion. The task was stopped if the participant grabbed for support. A pen attached to a rod and connected to a band around the participants’ waist was set to lie parallel to the ground and rest on a large paper grid (sway-meter). Sway was calculated as the largest distance covered across the grid.

### Functional Magnetic Resonance Imaging Acquisition

The MRI scans were conducted at the University of British Columbia (UBC) Hospital in Vancouver at the UBC MRI research center. A 3.0-Tesla Intera Achieva MRI Scanner with an 8-channel SENSE neurovascular coil was used. During the scanning procedure, the participants were told to rest with their eyes open, remaining completely still and thinking of nothing in particular for the duration of the session.

The scanning session consisted of an initial resting-state scan with 360 dynamic images of 36 slices (3 mm thick) with a repetition time (TR) of 2000 ms, an echo time (TE) of 30 ms, a flip angle (FA) of 90°, a field of view (FoV) of 240 mm and an acquisition matrix of 80 × 80. High-resolution anatomical T1 images were also acquired with 170 slices (1 mm thick), TR of 7.7 ms, TE of 3.6 ms, FA of 8°, FoV of 256 mm, and an acquisition matrix of 256 × 200.

### fMRI Pre-processing

FSL (FMRIB’s Software Library), MATLAB (Matrix Laboratory), and toolboxes from Statistical Parametric Mapping (SPM) were used to carry out image processing. The Optimized Brain Extraction Tool (optiBET) (Lutkenhoff et al., 2014) was used to remove any excess unwanted structures in high resolution T1 images, e.g., skull, eyes, etc. The rigid body motion correction was done using MCFLIRT with the absolute and relative mean displacement extracted and included as covariates in the statistical analysis. The Multivariate Exploratory Linear Optimized Decomposition into Independent Components (MELODIC) further removed any additional artifacts. FSL Motion Outliers was used to determine any data points that were corrupted with a large amount of motion. A confound matrix was used to remove the effects of these time points on any subsequent analyses. The Gaussian kernel of full-width-half-maximum (FWHM) 6 mm was used for spatial smoothing and temporal filtering was applied to create a signal between 0.008 < f < 0.08 Hz, which is the optimal range for analyzing resting-state functional connectivity data.

The functional MRI data was registered to the corresponding high resolution T1 anatomical image for each participant, which was then registered to standardized 152 T1 Montreal Neurological Institute (MNI) space. Regression of the cerebral spinal fluid, white matter and global brain signal was used to remove noise from any physiological or non-physiological sources. Finally, to account for the delay of hemodynamic response, the first four volumes of data were discarded.

### Functional Connectivity Analysis

The main focus of the functional connectivity analysis was to investigate the connectivity firstly within the DMN and secondly the inter-network connectivity between the DMN and the FPN and SMA independently. Previous studies guided the region of interest (ROI) selection for the analysis of the DMN, FPN, and SMA (Voss et al., 2010; Hsu et al., 2014). The ROIs within each network and their respective MNI space coordinates can be seen in **Table 1**. In order to analyze the overall interconnectivity between brain networks, the average of all the pairwise ROI–ROI correlations for each network was calculated. Preprocessed time-series data were extracted for each ROI with 14 mm diameter spherical ROIs drawn around their respective MNI coordinates in standard space. The time-series data for each ROI were then cross-correlated with every brain voxel to create functional connectivity maps of each neural network. Ordinary least-squares regression using FSL’s flameo (Beckman et al., 2003) was used to calculate group-level between subject results. The statistical map thresholding was set at *Z* = 2.33, with a cluster correction of *p* < 0.05.

**Table 1:** Region of interests (ROIs) with MNI coordinates for each network.

Network	ROI	*X*	*Y*	*Z*
DMN	PCC	8	–56	30
	FMC	–2	54	–12
	BMTG	58	–10	–18
		–52	–14	–20
	BPHG	24	–26	–20
		–26	–24	–20
	LMFG	–30	20	50
	BLOC	54	–62	32
		–44	–72	30
FPN	RIPS	25	–62	53
	BVV	36	–62	0
		–44	–60	–6
	RSMG	32	–38	38
	BLSOC	26	–64	54
		–26	–60	52
	BFEF	28	–4	58
		–26	–8	54
SMN	SMA	–5	–1	52

*DMN, default mode network; PCC, posterior cingulate cortex; FMC, frontal medial cortex; BMTG, bilateral middle temporal gyrus; BPHG, bilateral parahippocampal gyrus; LMFG, left middle frontal gyrus; BLOC, bilateral occipital cortex; FPN, frontoparietal network; RIPS, right inferior parietal sulcus; BVV, bilateral ventral visual; RSMG, right supramarginal gyrus; BLSOC, bilateral superior occipital cortex; BFEF, bilateral frontal eye field; SMN, sensorimotor network; SMA, supplementary motor area.*

### Data Analysis

Statistical analysis was conducted using the IBM SPSS Statistic 19 for Windows (SPSS Inc., Chicago, IL, United States). Descriptive data are reported for variables of interest. Alpha was set at *p* ≤ 0.05 for all analyses.

Partial correlations, adjusted for age and MoCA, were performed to investigate the association between: (1) dual task performance and DMN functional connectivity; and (2) measures of gait speed and postural sway and DMN functional connectivity

Additional partial correlational analyses were also conducted to determine whether functional between-network connectivity between the DMN–FPN and DMN–SMA were correlated with dual task performance, gait speed, and postural sway.

## Results

### Participants

A total of 40 participants were included in this study (see **Table 2**). However, five participants did not complete the dual task measure leaving 35 participants for analysis.

**Table 2:** Participant characteristics.

Variables		Mean (SD) or *n*
*N*		40
Female		21
Age (years)		76.75 (5.8)
Weight (kg)		74.16 (14.4)
MMSE (max 30)		27.50 (1.3)
MoCA (max 30)		22.30 (2.7)
Dual task cost	Single task time (s)	4.96 (1.4)
	Dual task time (s)	6.40 (2.7)
	Dual task cost	0.20 (0.2)
Gait speed (m/s)		1.17 (0.2)
Eyes open postural sway	Floor sway (mm)	64.48 (25.6)
	Foam sway (mm)	182.12 (87.4)

*MMSE, Mini-Mental Status Examination; MoCA, Montreal Cognitive Assessment.*

Based on visual inspection of the data there appeared to be a few outliers in the dual task cost results. Upon further analysis of all of the behavioral measures, two extreme outliers (mean ± 3 SD) were found in the dual task cost results. However, the removal of these outliers did not affect the results and as such, all data were included in the final analyses.

### Partial Correlations: Dual Task Performance, Gait Speed, Postural Sway, and DMN Functional Connectivity

**FIGURE 2:**
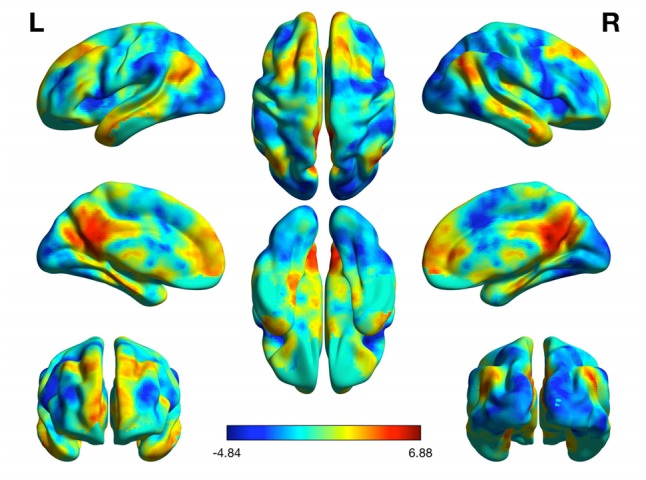
Resting state functional connectivity of the default mode network.

Partial correlation analyses showed a significant association between mean DMN functional connectivity and dual task cost (*r* = 0.427;*p* = 0.013), such that greater DMN connectivity was associated with poorer dual task performance. No other significant correlations were found (*p* ≥ 0.05). The resting state connectivity map for the DMN can be seen in **Figure 2**.

### Partial Correlations: Dual Task Performance, Gait Speed, Postural Sway, and Functional Between-network Connectivity between the DMN–FPN and DMN–SMA

**FIGURE 3:**
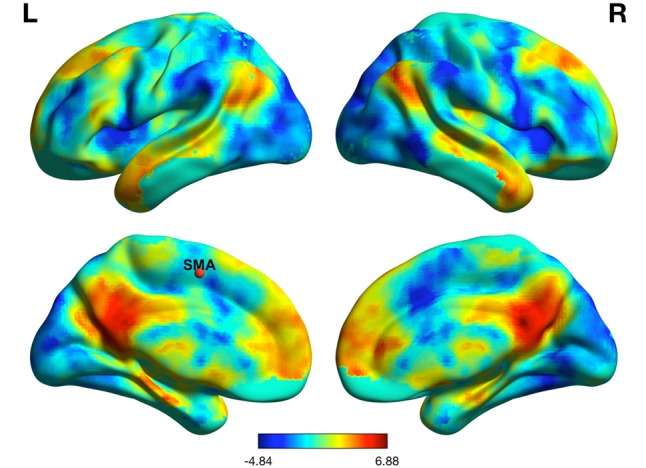
Resting state functional connectivity between the default mode network and supplementary motor area.

Mean resting state between-network DMN–SMA functional connectivity was significantly correlated with gait speed (*r* = -0.440; *p* = 0.01) as well as postural sway under the eyes open floor condition (*r* = 0.365; *p* = 0.037). Thus, greater between-network DMN–SMA functional connectivity was associated with slower gait speed and greater postural sway under the eyes open floor condition. Results of the partial correlation analyses are reported in **Table 3**. The resting state between network connectivity map for the DMN and SMA can be seen in **Figure 3**.

**Table 3:** Partial correlation results.

	Dual task cost	Gait speed	EO floor sway	EO foam sway
DMN	0.427^∗^	–0.125	0.075	–0.162
DMN–FPN	0.140	–0.108	–0.190	0.18
DMN–SMA	0.012	–0.440^∗^	0.365^∗^	0.36

*Adjusted for age and MoCA; ^∗^*p* < 0.05. EO, eyes open.*

## Discussion

We found that greater resting state DMN functional connectivity was significantly associated with greater dual task cost among community-dwelling older adults with MCI. It was previously discussed that greater DMN connectivity at rest is indicative of a lack of deactivation on task in older adults with MCI (Lustig et al., 2003; Mevel et al., 2011), which would subsequently result in a decrease in task performance. The association between greater DMN connectivity and reduced dual task performance found in this study is therefore likely evidence for a lack of deactivation of the DMN on task.

One possible explanation for greater dual task cost being associated with greater DMN connectivity is provided by the capacity sharing theory. This theory states that because attentional resources are limited, when a person is required to perform two attention-demanding tasks simultaneously, the performance of at least one of these tasks will deteriorate (Tombu and Jolicœur, 2003). Therefore, if the greater DMN connectivity at rest were evidence of a lack of deactivation on task, this would suggest that the DMN is providing additional competition for resources, reducing those available for task-related functioning and subsequently reducing performance on the task. In addition to competing for resources generally, the DMN would be using these resources to fuel a functional process responsible for increasing distraction from the task, further amplifying its negative impact on task performance.

Walking in everyday life is not performed as a single task. Hence, the dual task walking measure has been associated with replicating the demands of the environment whilst walking (Tideiksaar, 1996). Findings from the current study indicate that patients with MCI are unable to meet the cognitive demands required to maintain walking performance in everyday life due to a lack of resources available and reduced ability to maintain attention on the task. It is plausible that an increase in distraction whilst attempting dual task walking may contribute to the increase in falls risk seen in older adults with MCI.

In addition, we also found that the functional connectivity between the DMN and SMA was significantly associated with slower gait speed and increased postural sway. Given that the DMN would normally deactivate on task (Raichle et al., 2001), intact resting state DMN–SMA connectivity would usually be an indication that during task performance, when the SMA is active, the DMN is deactivated. In this case, greater connectivity between these brain regions at rest is negatively correlated with gait speed and thus suggests there is interference between these networks on task; most plausibly that the DMN is influencing the SMA by remaining active on task.

The SMA has been shown to play an active role in motor planning (Roland et al., 1980), and in maintaining attention on a motor task (Johansen-Berg and Matthews, 2000). Through the DMN remaining active, increasing competition for resources and reducing the ability to maintain attention on the task, the SMA is unable to create motor plans with as much accuracy or efficiency. This would likely result in reduced quality of gait control and subsequently, gait speed. In addition, this decline in gait speed will further contribute to an increased risk of falls (Verghese et al., 2009; Espy et al., 2010). It would be useful to establish whether interventions targeted at increasing gait speed are also found to alter this maladaptive connectivity in order to provide support for this theory.

The SMA has also been implicated in postural control. Findings from Viallet et al. (1992) lead to the suggestion that the SMA was responsible for selecting the relevant circuits of phasic postural adjustments in order to maintain posture. Consequently, if an increase in DMN activity on task were to add competition for resources to the SMA, it is likely that there would be a decrease in the ability to select the correct postural adjustments to maintain balance, leading to the increased sway evident in this study.

It is important to note that greater connectivity between the DMN and SMA only correlated with performance on the floor sway task and not performance on the foam sway task. It is thought this is because the foam sway task may require more overt attention. Whereas, the participants may be less inclined to intentionally focus on the floor sway task because they are not so obviously unstable. This may be better explained by the capacity model of attention, which states that when a task is less cognitively demanding, more resources remain available to be allocated to task-irrelevant networks (Kahneman, 1973). In this case, the additional resources are allocated to the DMN. It may therefore be that when performing the foam sway task, participants were able to overcome the DMN interference by actively allocating more resources to the task-related regions than when performing the floor sway task. However, this can only be considered a speculative explanation as participants were not asked about how much attention they paid to the task and task-related neuronal activity could not be measured. This theory is also contradictory to the capacity sharing theory that has been used to explain the findings in relation to dual task performance, known to be a cognitively demanding task. Investigating the effect of the DMN–SMA connectivity over progressively more challenging postural tasks would be beneficial to determine which theory is most likely to explain these results.

There was no relationship found between connectivity of the DMN and FPN at rest and any of the behavioral measures. Due to the lack of previous literature in this area it is not completely clear why this may be. One of the studies that did find increased connectivity between the DMN and FPN during the performing of a motor task, found the increase in connectivity was specific to older adults classified as fallers rather than non-fallers (Hsu et al., 2014). Consequently, it may be that the FPN is more resistant to the influence of the DMN until a later stage in mobility decline. Further research is required to investigate the effect of the DMN on the FPN across several stages of falls risk, ideally comparing healthy older adults to those with MCI and those classified as fallers in order to establish at what stage connectivity between the DMN and FPN may become detrimental, if at all.

This study only compared resting state brain network connectivity with behavioral measures and did not investigate brain activity on task. Due to the limitations of functional MRI (fMRI) scanning (i.e., sensitivity to movement artifacts), and the lack of MRI-safe apparatus it was not possible for us to assess activity of the DMN and associated networks while performing motor tasks such as walking. Thus, caution must be taken when interpreting these results, as they are only correlational. To support our findings, future studies should aim to investigate the functional connectivity both within and between these networks whilst simultaneously performing a motor task. It is also important to acknowledge that there may be other underlying factors that could account for these results. For example, this study did not investigate the impact of potential sex differences or the underlying structural integrity of these networks, which may have provided an alternative explanation of the findings. Consequently, future studies should aim to identify any sex differences and compare both the structural and functional associations between these networks and mobility factors in order to better understand the underlying neurobiological factors contributing to increased falls risk. In addition, our study consisted of community-dwelling older adults with MCI exclusively and we did not have ethical clearance to collect any data pertaining to the race or ethnicity of our participants, as this was not related to our hypotheses. Thus, we acknowledge that this may make the generalizability of our findings ambiguous. Furthermore, some evidence highlights the potential influence of the cerebellum on the networks discussed in this study (Habas et al., 2009). Although this was not one of the main focuses of the current study, it may be beneficial to additionally investigate the role of the cerebellum in relation to these networks in future research. Due to the exploratory nature of this study and the small sample size, no adjustments were made to the significance value in order to control for multiple comparisons. It is acknowledged that this increases the likelihood of a type I error and future studies with larger sample sizes are needed to confirm our current findings. Finally, walking was performed indoors on a GAITRite mat and a verbal subtraction task was used for the dual task, consistent with other studies using a dual task walking paradigm (Beurskens and Bock, 2012). However, it would be useful to determine the specific effects of other cognitive and motor tasks whilst walking over varying terrains as well, as this is likely to be more representative of real-world scenarios.

Our results show that increased resting state DMN connectivity is associated with a decrease in dual task performance, slower gait speed and increased postural sway in older adults with MCI; either via greater within network connectivity or between-network connectivity with the SMA. This supports the theory proposed initially in **Figure 1**. To our knowledge, this is the first study to investigate the role of the DMN on dual task performance and motor functioning in people with MCI. These findings can be used to determine if interventions targeted to improve gait, cognition, and/or dual tasking specifically, can reduce the maladaptive effects of greater DMN connectivity in older adults vulnerable to an increased risk of mobility disability.

## Author Contributions

RC, TL-A, and CLH were involved in designing and performing the study. All authors contributed to the data analysis. RC, TL-A, and CLH were involved in the interpretation of results. RC wrote the first draft of the manuscript. TL-A, JB, and CLH wrote portions of the manuscript and critically reviewed the manuscript. All authors have read and approved the manuscript.

## Conflict of Interest Statement

TL-A is a Canada Research Chair in Physical Activity, Mobility and Cognitive Neuroscience. JB is a Canadian Institutes of Health Research and Michael Smith Foundation for Health Research Postdoctoral Fellow. CLH is an Alzheimer Society Research Program Doctoral Trainee. The other author declares that the research was conducted in the absence of any commercial or financial relationships that could be construed as a potential conflict of interest.
